# 

**Published:** 1900-11-17

**Authors:** 


### NOTICE TO CORRESPONDENTS.

All MSS., Letters, Books for Review, and other matters intended for the Editor should be addressed THE EDITOR, "THE HOSPITAL," THE HOSPITAL BUILDING, 28 & 29 SOUTHAMPTON STREET, STRAND, W.C.

The Editor cannot undertake to return rejected MSS., even when accompanied by stamped directed envelope.

**Notice to Advertisers and Subscribers.**

All Advertisements, Orders for Copies of THE HOSPITAL, and Business Communications should be addressed to **THE MANAGER (Not to the Editor)**, THE HOSPITAL, 28 & 29 Southampton Street, Strand, London, W.C.

The Cost of Subscription, post free, is as follows:—Per week, 2½d.; per quarter, 2s. 8d.; per half-year, 5s. 3d.; per annum, 10s. 6d. Foreign:—Per annum, 15s. 2d., payable, in all cases, in advance.

**NOTICE.—Vol. XXVIII. of "The Hospital" (April to September, 1900), in handsome cloth binding, is now on sale at the Office of this Journal, price, 7s. 6d. Cases for binding the Numbers contained in this Volume 1s. 6d. Index to Vol. XXVIII., 2½d., post free.**

**The Monthly Part for November is now ready. Price 6d., by post 8d.**

### Scraps and Gleanings.

The total income for the past year of the Dumbarton Public Eye Dispensary amounted to £180 odd, and the expenditure to £192. The balance of income from the previous year amounted to £48, so that the institution has a balance to the good of £44 odd.

The amount handed over this year to the Coventry and Warwickshire Hospital as the result of the twenty-seventh annual appeal of the Coventry Working Men's Hospital Saturday Committee, was £1,250, being £15 larger than that of last year.

As a result of the special efforts of the authorities to put the finances of the Hereford Infirmary in a more satisfactory condition, the chairman was able to report at the recent quarterly meeting of the Governors that, thanks to the initiative of the Mayor, £1,500 had already been received towards the deficiency.

At the annual meeting of the Greenock Eye Infirmary, the medical officer in his report directed attention to the large number of school children who were annually treated, and stated that much unnecessary annoyance and trouble were caused by the over vigilance of the School Board officers, who by threats of prosecution, got the children to attend school to the neglect of their eyes.

The receipts for the last year of the Alnwick Infirmary were £405 and the expenditure £428. These receipts show a decrease, as compared with those for the previous year, of £73, the expenditure having also decreased by about £50.

A flower stall which was held in Aylsham Market Place for a few hours once a week during July, August, and September has resulted in £7 13s. 4d. being handed to the Norfolk and Norwich Hospital. This amount represents the nett proceeds from the sale of the flowers.

The total cost of the new Union Infirmary at Edmonton, which was recently opened, will be about £22,000. The new buildings provide accommodation for 150 patients, and are intended for acute cases, the chronic cases being kept in the old wards. There are to be 27 nurses attached to the new infirmary.

At a recent fortnightly meeting of the Ormskirk Board of Guardians, on the ground that the meeting which passed the resolution was not representative of the whole Board, the resolution passed in June last, substituting the scattered cottage home system for the present industrial schools in the treatment of pauper children, was rescinded, the motion being carried by 23 votes to 17.

The fourth day of the Newport Hospital Bazaar proved as great a success from the monetary point of view as the preceding ones, the sum received being £1,184. Mr. F. Davis promised to add a cheque of £150 for every £1,000 or portion of £1,000 realised. As the four days' takings amounted to over £4,000, his gift will amount to £750, making a gross total realised by the bazaar of £5,451 odd.

The total receipts in connection with this year's Guildford Hospital Saturday collection were £200 13s. 7d., the expenses amounting to £9 17s. The hon. secretary of the Hospital Saturday Committee said that this was a record collection in many ways, for never before had the receipts amounted to £200, nor had they ever before had a nett balance of £190. This amount was divided as follows:—Royal Surrey County Hospital, £93; Surrey Convalescent Home, £62; Victorian Convalescent Home for Surrey Women, £31; leaving a balance in the bank of £4 16s. 2d.

### The Student of Medicine.

### APPOINTMENTS.

Wilfred E. Alderson, M.D., M.S.Durh., appointed Surgeon to Trinity House, Newcastle-on-Tyne. Lullum Wood Bathurst, M.D.Lond., appointed Assistant Medical Examiner to the Technical Education Board of the London County Council. John Archibald Campbell, L.R.C.P. & S.Edin., L.F.P. & S.G., appointed Demonstrator of Anatomy at Anderson's College Medical School, Glasgow. L. F. Corbet, L.R.C.P., L.R.C.S.Irel., appointed Certifying Factory Surgeon for the Killoughy District in the Union of Tullamore. Hugh Davies, F.R.C.S.Eng., M.B., B.S.Lond., appointed Surgeon to the Miller Hospital, Greenwich. Frederic Durham, M.B., F.R.C.S., appointed Consulting Surgeon to the North-West London Hospital, Kentish Town Road, N.W. Lachlan Grant, M.D.Edin., appointed Parish Medical Officer and Public Vaccinator for Ballachulish and Glencoe; also Certifying Factory Surgeon for Parish of Lismore and Appin, Argyleshire. B. Burnett Ham, M.D., M.R.C.S., L.R.C.P., D.P.H.Camb., appointed Commissioner of Public Health for Queensland, Australia. Nathaniel H. Hobart, B.A., M.B., B.C.Cantab., appointed Intern Surgeon to the North Charitable Infirmary, Cork. John James Huey, L.S.A., appointed Medical Officer and Public Vaccinator for the Mexborough District of the Mexborough Union. F. J. Macguire, L.R.C.P., L.R.C.S.Irel., appointed Medical Officer for the Longford Workhouse. I. Macquarrie, M.B., C.M., appointed Medical Officer for the Ireby District of the Wigton Union. Noel Maudsley, M.B., C.M.Edin., appointed Medical Officer for the Alwinton District of the Rothbury Union. Reginald S. Nichol, M.B., Ch.B.Vict.Univ., appointed House-Surgeon to the Southern Hospital, Manchester. A. G. Robb, M.B.R.U.I., appointed Superintendent Medical Officer to the Fever Hospital, Belfast Union. Frank Thomas, M.B., B.C.Cantab., appointed Ophthalmic Surgeon to the Swansea Hospital.

The following appointments have been made at the Leeds General Infirmary:—Mr. H. Collinson, M.R.C.S., L.R.C.P., to be Resident Medical Officer at the Ida Hospital. Mr. E. W. Anderton, M.B., Ch.B. (Vict.), to be Resident Obstetric Officer. Mr. W. Gough, M.R.C.S., L.R.C.P., to be one of the House Surgeons. Mr. H. C. Alderman, M.R.C.S., L.R.C.P., ditto. Mr. J. Lückhoff, M.B., C.M. (Ed.), ditto.

